# Surgical treatment pattern and outcomes in epithelial ovarian cancer patients from a cancer institute in Kerala, India

**DOI:** 10.3332/ecancer.2016.619

**Published:** 2016-02-04

**Authors:** P Georgeena, Anupama Rajanbabu, DK Vijaykumar, K Pavithran, KR Sundaram, KS Deepak, MR Sanal

**Affiliations:** 1 Public Health Research Department, Amrita Institute of Medical Sciences, Kochi, Kerala 682041, India; 2 Department of Gynaecologic Oncology, Amrita Institute of Medical Sciences, Kochi, Kerala 682041, India; 3 Department of Medical Oncology, Amrita Institute of Medical Sciences, Kochi, Kerala 682041, India; 4 Department of Biostatistics, Amrita School of Medicine, Amrita Institute of Medical Sciences, Kochi, Kerala 682041, India; 5 Clinical Research Department, Amrita Institute of Medical Sciences, Kochi, Kerala 682041, India

**Keywords:** advanced epithelial ovarian cancer, recurrence-free survival, primary surgery, NACT

## Abstract

**Objective:**

To evaluate the treatment and survival pattern of patients with advanced epithelial ovarian cancer.

**Methods and results:**

Retrospective study of all advanced epithelial ovarian cancer patients treated in the department of gynaecologic oncology from an academic centre, in a four year period from 1 January 2008–31 December 2011.

**Selection criteria:**

All patients with advanced epithelial ovarian cancer (stage III and IV) who underwent surgery from 2008–2011and had a follow-up of at least three months after completion of treatment were included. The decision on whether primary surgery or neoadjuvant chemotherapy (NACT) in advanced ovarian cancer was based on age, performance status, clinical and imaging findings.

**Results:**

A total of 178 cases of epithelial ovarian cancer were operated on during this four year period. Among them 28 patients were recurrent cases, 22 had early stages of ovarian cancer, and the rest 128 had stage III and IV ovarian cancer. In these 128 patients, 50(39.1%) underwent primary surgery and 78(60.9%) had NACT followed by surgery. In the primary surgery group 36(72.0%) patients had optimal debulking while in the NACT group 59(75.6%) patient had optimal debulking. With a median follow-up of 34 months, the median overall survival (OS) and progression free survival (PFS) was 53 and 49 months respectively. Patients who underwent primary surgery had better median PFS than patients who had NACT (56 months versus 39 months, p = 0.002). In stage III C the difference median PFS was significant for those treated with primary surgery when compared with NACT (55 months versus 39 months, p = 0.012). In patients who had optimal debulking to no residual disease (n = 90), primary surgery gave a significant improved PFS (59 months versus 38 months, p = 0.001) when compared with NACT. In univariate analysis, NACT was associated with increased risk of death (HR: 0.350; CI: 0.177–0.693).

**Conclusion:**

In advanced epithelial ovarian cancer, primary surgery seems to have a definite survival advantage over NACT in patients who can be optimally debulked to no residual disease.

## Background

Ovarian cancer is the most deadly of all gynaecological cancers. Even though new treatments and newer chemotherapeutic agents have been introduced, the five year survival is low, especially in India [1–4]. It ranks second position in the most common gynaecological cancer among Indian women [5]. The current standard of treatment for epithelial ovarian cancer (EOC) is complete cytoreductive surgery (CRS) to remove the primary tumour and debulking of any metastatic disease combined with systemic chemotherapy using paclitaxel and platinum-based agents (carboplatin/cisplatin), though two recent randomised trials have shown that initial chemotherapy followed by interval debulking surgery is not inferior [6, 7]. One of the most important factors affecting survival is the amount of residual tumour after surgery [8, 9]. In patients who cannot be optimally debulked, NACT (neoadjuvant chemotherapy) followed by interval debulking is the treatment of choice.

## Methods

We included all patients who underwent surgery for advanced epithelial ovarian cancer from the Department of Gynaecologic Oncology, over a four year period from 1 January 2008–31 December 2011 at the Amrita Institute of Medical Sciences (AIMS) Kerala, India. Approval was obtained from the Institutional Review Board (Reg:ECR/129/Inst/KL/2013) and data were retrieved from the patient’s records and institutional cancer registry. Staging was done using the Federation of Gynaecology and Obstetrics, FIGO (1989) classification of ovarian cancer. Patients were selected for primary surgery based on age, performance status, and imaging features. The residual disease were quantified as optimal to no residual disease, optimal to less than 1 cm and suboptimal as during the study period where optimal debulking was considered as residual disease <1 cm. The chemotherapy used was paclitaxel (175 mg/m^2^) and carboplatin with an area under curve (AUC) 5. In patients undergoing primary surgery six cycles of paclitaxel with carboplatin was given as adjuvant treatment. The NACT group received three cycles in the neoadjuvant setting and three cycles as adjuvant treatment. Median overall survival (OS) and progression free survival (PFS) was estimated by Kaplan-Meier method and comparison of the groups was done using the log-rank test. A multivariate Cox-regression analysis was done to estimate the hazard ratio (HR) and 95% confidence interval (CI) for the different variables. The comparison was done using the chi-square test. The data were analysed using IBM SPSS (V.20.0). P-Values < 0.05 were considered statistically significant.

## Results

A total of 178 patients underwent surgery for epithelial ovarian cancer from AIMS, out of which 28 patients were recurrent cases, 22 had early stages of ovarian cancer, and the remaining 128 had stage III or IV ovarian cancer. Among the 128 patients, 75(58.6%) patients had complete treatment from AIMS but the rest 53(41.4%) patients had partial treatment from AIMS. Distribution details are given in Table 1. In 128 patients with stage III or IV ovarian cancer were 101(78.9%). Among them 78(60.9%) of them underwent NACT and the rest 50(39.1%) had primary surgery. The decision for primary surgery versus NACT was based mainly on the assessment by the surgical team of the possibility of optimal debulking and ability to withstand a prolonged surgery. Advanced age, poor performance status, and poor nutrition were factors which posed significant risk for prolonged anaesthesia and major surgery, and hence such patients were taken for NACT and interval debulking.

**Figure 1.**
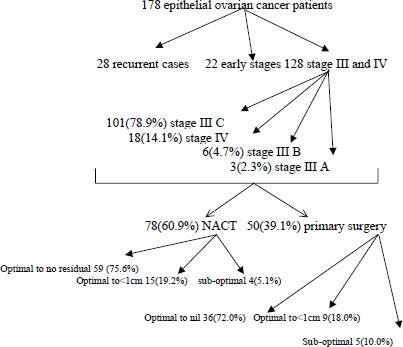
Results.

**Figure 2.**
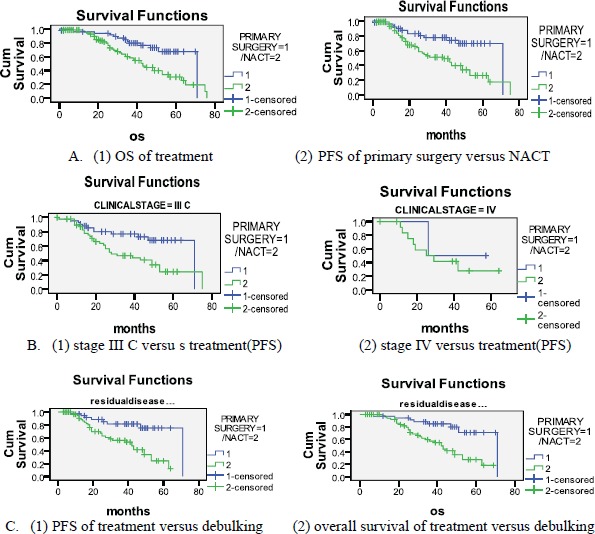
Kaplan-Meier survival curves.

Among the 50 patients of stage IIIC and IV who underwent primary surgery, we were able to obtain optimal debulking in 90% (with 72% being optimal debulking to no residual disease). In the five patients who did not have optimal debulking, there was disease involving porta hepatis and small bowel mesentery in two patients, whereas three patients could not be debulked because of haemodynamic instability. Among the 78 patients who underwent NACT, four (5.1%) patients had suboptimal surgery. This was because of disease involving bowel mesentry and haemodynamic instability. (Figure 1) (Table 2) with a median follow-up of 34 months, 23(59%) patients had recurrence in the NACT group, whereas patients who had undergone primary surgery, disease recurred in only 16(41%). The median OS and PFS time for all patients was 53 months and 49 months respectively after a median follow-up of 34 months. The primary surgery group had PFS of 56 months versus 39 months in the NACT group which was statistically significant (p = 0.002) (Figure 2A) and

OS 58 and 44 months respectively (p = 0.002). On subset analysis it was seen that in stage IIIC and IV patients the median OS was 53 months and the median PFS was 49 months (n = 119). Those stage IIIC patients who underwent primary surgery had better survival than the NACT group (PFS = 55 months versus 39 months, p = 0.012). The median OS and PFS for optimal debulking were 51 months and 49 months respectively. When OS was compared between optimally debulked patients, the primary surgery group had significantly improved survival rates (PFS = 59 months versus 38 months, p = 0.001) (Figure 2B). In stage III C and IV, the primary surgery patients with optimal debulking to no residual disease had significantly higher survival than those who were treated with NACT (58 months versus 39 months, p = 0.004) (Figure 2C, Table 1). The PFS for those patients who completed treatment in AIMS was significantly higher (53 months versus 43 months) than those who had partial treatment outside AIMS.

In the univariate analysis the NACT group had a higher risk for death, 65% when compared with primary surgery group (HR: 0.350; CI: 0.177–0.693).

## Discussion

This retrospective study was done with the aim of studying the treatment pattern and survival of advanced EOC in Indian women. We found that more patients were given NACT than primary surgery but primary surgery was associated with better outcomes within a four year follow-up period. The difference was statistically significant for Stage IIIC in optimally as well as sub-optimally debulked patients.

The disease-free survival (DFS) for the NACT group was 39 months and 35 months respectively for those belonging to stage IIIC and IV, whereas in primary surgery it was 55 months and 41 months. Studies have shown that NACT improved the survival in stage IV patients [10, 11] whereas our study showed no significant survival advantage for stage IIIC and IV patients with NACT (p = 0.544). This result may be because of the selection bias of the majority of advanced stage patients who were selected for NACT (n = 73). In a Danish study done by Fago-Olsen *et al,* there was no significant difference in the median OS between the primary surgery and NACT groups, whereas the risk of death after two year follow-up has been increased in NACT group [12]. A previous Indian study by Deo SVS *et al* with a median follow-up of 34 months, reported the median overall and five year DFS as 31 and 32% respectively for stage IIIC and IV patients. The median disease-free interval was 25.4 months [13]. Hence our study shows that primary surgery has good prognosis when compared with NACT in stage IIIC and IV patients. The standard treatment for FIGO stage III and IVA ovarian cancer tends to be primary surgery but no randomised controlled trials have yet been done to prove that prognosis is better with primary surgery [14].

Several studies have shown that surgical cytoreduction followed by platinum-based chemotherapy is the backbone for the treatment of advanced EOC but NACT and interval debulking have equally evolved [8, 9, 15–17]. NACT is also selected for patients with poor performance and those have large un-resectable tumour [18]. In our study the OS and DFS in advanced EOC was found to be 53 and 49 months respectively (median follow-up of 34 months). There are studies showing that the median OS and PFS for advanced EOC are 26 and 18 months respectively for interval debulking patients [19].

The OS and PFS were 58 and 56 months for primary surgery, 44 and 39 months in NACT (median follow-up 34 months) in our study. This study concluded that the primary surgery is associated with better prognosis and studies by Bristow *et al* have found that NACT is associated with worse prognosis when compared with primary surgery [20]. In our study there was a significantly improved OS in the optimally debulked primary surgery group. According to European Organisation of Research and Treatment of Cancer—National Cancer Institute of Canada (EORTC–NCIC) study, the removal of all macroscopic disease during primary surgery has been the most important independent prognostic factor in advanced ovarian cancer [21]. The point to be noted is that, even though an increased percentage of patients (75.6%) had optimal debulking in NACT group this did not account for a better survival. These results found to be similar to the Danish Study by Fago-Olsen *et al* [12]. We also found that in primary surgery group with stage III C and IV who had optimal debulking, the survival rates tend to be higher than NACT group.

The main limitations of our study are that it was retrospective series and it was not a randomised control trial. As it is an un-randomised study the selection bias affects the OS. Another problem faced during the study was tracking the patients lost to follow-up. Here the efficiency of the cancer registry system comes into picture which has helped in tracking the patients. Our study also showed that among the 128 patients, 58.6% have completed their treatment in AIMS but the remaining 41.4% have not completed their treatment in AIMS. This may be because of the poor financial status faced by the Indian population; they may opt for a health care setting which provides treatment at lower cost. The accessibility of the advanced treatment modalities is low in the more rural areas and in order to receive the treatment they may have to travel to the urban areas which may in turn compel the patients to drop the treatment. India provides most of the essential drugs, especially anticancer drugs at low cost which are affordable, even then there are patients who are unable to purchase medicines at this cost. Thus the need for cost effective and efficient treatment should be adopted in a developing country like India.

Another problem faced was the selection criteria of patients for primary surgery and NACT. Even though there are different selection criteria available, there are no standardised criteria for common use. We selected patients based on performance status and disease distribution as seen by CT imaging, but still optimal debulking to no residual disease was only 72% in the primary surgery group. We need better modalities to predict debulking and of late we have started using diagnostic laparoscopy to assess whether the patient can be optimally debulked or not.

## Conclusion

Despite aggressive treatment with surgery and chemotherapy, EOC patients still have poor OS. Primary surgery, if it achieves optimal debulking to no residual disease, has an advantage over NACT in advanced EOC. Identifying these groups of patients still remains a challenge. Research need to focus on the pre-operative identification of optimally debulkable advanced ovarian cancer so that the benefit of primary surgery can be extended to them while sparing the morbidity of initial surgical exploration in the group that cannot be optimally debulked.

## Conflict of interest statement

The authors declare that there are no conflicts of interest.

## Funding statement

The funding for this study was provided by Amrita Institute of Medical Sciences, Kochi.

## Figures and Tables

**Table 1.** Overall and progression-free survival.

		OS (months)	PFS (months)	n
Primary surgery		58	56	128
NACT		44	39	128
Stage IIIC and IV		53	49	119
Optimal debulking		51	49	128
Treatment:	In AIMS	71	53	75
	Outside AIMS	47	43	53

	OS (months)			PFS (months)			n
	Primary surgery	NACT	p-value	Primary surgery	NACT	p-value	
Stage III C	57	46	0.021	55	39	0.012	101
Optimal debulking	60	42	<0.001	59	38	0.001	95

**Table 2.** Distribution of stage, residual disease, and treatment regimen.

	STAGE					RESIDUAL DISEASE			
	III A	III B	III C	IV	TOTAL	Optimal to Nil	Optimal to <1 cm	Sub-optimal	TOTAL
**Primary surgery**	0 (0%)	4 (8.0%)	44 (88.0%)	2 (4.0%)	50 (100%)	36 (72.0%)	9 (18.0%)	5 (10.0%)	50 (100%)
**NACT**	3 (3.8%)	2 (2.6%)	57 (73.1%)	16 (20.5%)	78 (100%)	59 (75.6%)	15 (19.2%)	4 (5.1%)	78 (100%)
**TOTAL**	3 (100%)	6 (100%)	101 (100%)	18 (100%)	128 (100%)	95 (74.2%)	24 (18.8%)	9 (7.0%)	128 (100%)
